# Neuroimaging Results, Short-term Assessment of Psychomotor Development and the Risk of Autism Spectrum Disorder in Extremely Premature Infants (≤28 GA) - a Prospective Cohort Study (preliminary Report)

**DOI:** 10.34763/devperiodmed.20182201.3948

**Published:** 2018-04-12

**Authors:** Magdalena Rutkowska, Monika Bekiesińska-Figatowska, Grażyna Kmita, Iwona Terczyńska, Katarzyna Polak, Marcin Kalisiak, Dorota Prażmowska, Eliza Kiepura, Sylwia Szkudlińska-Pawlak, Małgorzata Seroczyńska, Ewa Helwich

**Affiliations:** 1 Klinika Neonatologii i Intensywnej Terapii Noworodka Instytut Matki i Dziecka, Warszawa, Polska; 2 Zakład Diagnostyki Obrazowej Instytut Matki i Dziecka, Warszawa, Polska; 3 Wydział Psychologii, Uniwersytet Warszawski, Zakład Wczesnej Interwencji Psychologicznej Instytut Matki i Dziecka, Warszawa, Polska; 4 Klinika Neurologii Dzieci i Młodzieży Instytut Matki i Dziecka, Warszawa, Polska; 5 Poradnia Okulistyczna Instytut Matki i Dziecka, Warszawa, Polska

**Keywords:** prematurity, follow-up, autism, neuroimaging studies, noworodek przedwcześnie urodzony, ocena rozwoju, autyzm, badania neuroobrazowe

## Abstract

Infants ≤28 GA are at particular risk of psychomotor and neurological developmental disorder. They also remain at a higher risk of developing autism spectrum disorder (ASD), characterized by persistent deficits in communication/social interactions and restricted, repetitive behaviors, activities and interests. Monitoring their development by a team of specialists (a neurologist, psychologist, psychiatrist) allows us to make an early diagnosis and to implement appropriate therapy. Neuroimaging studies during the neonatal period may be helpful in clarifying diagnosis and prognosis.

**Objective:**

The aim of the study was to search for the interrelation between the results of neuroimaging and the neurological, psychological and psychiatric evaluation at the age of 2.

**Material and methods:**

Neonates born at ≤28 weeks between 01.06.2013 and 31.12.2015 and hospitalized at NICU were enrolled. We present the results of the first 12 children who have attained 2 years of corrected age and have undergone both neuroimaging, and neurological, psychological and psychiatric assessments. Transfontanel ultrasound was performed according to general standards, MRI between 38 and 42 weeks of corrected age. Neurological examination based on the Denver scale, ASD screening with use of the STAT test and psychological DSR assessment were performed at 2 years of corrected age.

**Results:**

Median GA was 26 weeks and median weight 795 g. The ultrasound examination was normal in 9 cases (75%) and MRI in 4 (33%). Abnormalities in the cerebellum were the main additional information found in MRI as compared to US. Neurological examination was normal in 8 infants (67%), in 4 of whom neuroimaging was normal. In 4 (33%) infants the neurological examination was abnormal. Psychomotor development at an average level or above was found in seven (58%) children. In 4 of them neuroimaging was normal, whereas 3 had ventricular dilatation and haemorrhagic infarct. There were no abnormalities within the cerebellum in this group. In the remaining 5 children (42%) psychomotor development was rated as delayed. All of them had cerebellar haemorrhage. An increased risk of ASD was observed in 4 children who developed cerebellar hemorrhage.

**Conclusions:**

1. The use of MRI at a term-equivalent age may contribute to the prognosis of neurodevelopmental outcomes in extremely premature infants, allowing risk stratification and thus enhancing early monitoring of a child’s development and functional status 2. There is a clear tendency towards abnormal psychomotor development and positive screening for ASD to co-occur with abnormal MRI findings in the cerebellum.

## Streszczenie

Noworodki skrajnie niedojrzałe (≤28 tc) są grupą dzieci szczególnie narażoną na zaburzenia rozwoju psychoruchowego, istnieje też u nich większe ryzyko wystąpienia zaburzeń ze spektrum autyzmu (ASD) charakteryzujących się zaburzeniami komunikacji/interakcji społecznych oraz stereotypowymi, powtarzalnymi zachowaniami. Monitorowanie rozwoju przez zespół specjalistów (neurolog, psycholog, psychiatra) pozwala na wczesne rozpoznanie i wdrożenie odpowiedniej terapii. Badania neuroobrazowe wykonywane w okresie noworodkowym mogą być pomocne w postawieniu dokładniejszego rozpoznania i w przewidywaniu rokowania.

**Cel:**

Celem badania było poszukiwanie związku między wynikami badań neuroobrazowych a oceną neurologiczną, psychologiczną i psychiatryczną dzieci w wieku 2 lat.

**Materiał i metody:**

Badaniem objęto noworodki urodzone ≤28 tc w okresie 01.06.2013-31.12.2015 i hospitalizowane w Klinice Neonatologii i Intensywnej Terapii Noworodka Instytutu Matki i Dziecka. Przedstawiamy wyniki pierwszych 12 dzieci, które osiągnęły wiek korygowany 2 lat i u których wykonano zarówno badania obrazowe, badanie neurologiczne, jak i ocenę psychologiczną i psychiatryczną. USG p/ciemiączkowe wykonywano wg ogólnych standardów, badanie przy użyciu rezonansu magnetycznego (MR) między 38 a 42 tygodniem wieku postkoncepcyjnego. Badanie neurologiczne w oparciu o skalę Denver, przesiewowe w kierunku ASD z wykorzystaniem testu STAT i ocenę psychologiczną Dziecięcą Skalą Rozwojową (DSR) wykonano w wieku korygowanym 2 lat.

**Wyniki:**

Średni wiek ciążowy wynosił 26 tc, a średnia masa ciała 795 g. Wynik badania USG był prawidłowy w 9 przypadkach (75%), a MR w czterech (33%). Główną dodatkową informacją stwierdzaną w MR w stosunku do badania USG były nieprawidłowości w obrębie móżdżku. Wynik badania neurologicznego nie odbiegał od normy u 8 dzieci (67%), u 4 z nich neuroobrazowanie było również prawidłowe. U pozostałej 4 dzieci (33%) wynik badania neurologicznego był nieprawidłowy. Co najmniej przeciętny wynik DSR stwierdzono u 7 dzieci (58%): u 4 z nich badania neuroobrazowe były prawidłowe, u 3 stwierdzono pokrwotoczne poszerzenie komór i zawał krwotoczny. W tej grupie nie stwierdzono nieprawidłowości w obrębie móżdżku. U pozostałych 5 dzieci (42%) poziom rozwoju psychoruchowego wskazywał na opóźnienie rozwoju i u nich wszystkich stwierdzono krwawienie do móżdżku. Zwiększone ryzyko ASD stwierdzono u 4 dzieci, u których także uwidoczniono krwawienie do móżdżku.

**Wnioski:**

1. Użycie rezonansu magnetycznego (MR) zwiększa możliwości rokownicze i może mieć ważne znaczenie dla monitorowania wczesnego rozwoju tej populacji dzieci 2. Rysuje się wyraźna tendencja do powiązania nieprawidłowego rozwoju psychoruchowego i przesiewowego w kierunku ASD z nieprawidłowymi wynikami badania MR w obrębie móżdżku.

## Introduction

Extremely premature infants (born ≤28 weeks of gestation [GA], body weight <1000 g) are a high risk group for abnormal physical and psychomotor development. Among the major problems requiring detailed assessment are: growth, respiratory and vision disorders, motor and cognitive development. This is due to the fact that severe neurodevelopmental complications (cerebral palsy and cognitive developmental delays) that appear between 2 and 3 years of age, affect 12-18% of this population. Neurodevelopmental and psychological disorders, such as behavioral, cognitive and emotional disturbances, appear until school age, and even beyond. Autism spectrum disorder is observed in these children over 10 times more oThen than in the population of term neonates (8% vs. 0. 6%). Vision problems (including myopia, strabismus), especially in children with retinopathy of prematurity, affect between 20 and 50% of this population. This is indicated both by world research and the few studies conducted in Poland [1, 2, 3, 4, 5, 6, 7].

In addition to clinical examination, a significant role is assigned to neuroimaging studies in evaluating abnormalities and predicting prognosis in this population of children. There is no doubt that cranial ultrasound is and will remain the primary diagnostic tool as a non-invasive, easily accessible and inexpensive technique. However, it has significant limitations, so nowadays magnetic resonance imaging (MRI) is the best way to visualize the brain and the necessary technique for evaluating changes within it. MRI allows the visualization of the posterior fossa structures (cerebellum, brainstem), of the cortex and deep structures of cerebral hemispheres, including the thalami and basal ganglia, as well as for the assessment of myelination. Meta-analysis of the first world studies seeking correlations between ultrasound examinations performed during hospitalization, brain MRI in the 40th week of postconceptional age and neurological examination evaluating the long-term development of extremely prematurely-born babies indicates the important role of MRI in determining prognosis [9, 10, 11, 12].

With regard to cognitive development and functioning, extreme prematurity is a risk factor for learning dificulties, even in children with a normal level of intellectual development [13]. Possible and coinciding causes of these dificulties are deficits in executive functions [14], phonological processing [15], but also attention deficit hyperactivity disorder (ADHD), predominantly the inattentive type. Internalizing disorders, especially anxiety disorders, also play an important role [15]. In addition, an eightfold increase in the risk of autism spectrum disorders (ASD) [16] has been observed in extremely prematurely born infants compared to the term infant population. According to the Diagnostic and Statistical Manual of Mental Disorders, 5^th^ edition (DSM-5), ASD is a complex neurodevelopmental disorder covering a broad range of deficits and symptoms in two major domains: 1) persistent deficits in social interaction and communication across multiple contexts, 2) restricted, repetitive patterns of behavior and activities, as well as unusual interests.

Among potential causes or factors correlating with ASD in this population (apart from genetic predispositions and primary and secondary infections), cerebellar and thalamic abnormalities are generally noted, which are visible on MR imaging only [17]. In addition, synaptogenesis dysfunction is also considered in the etiopathogenesis of various disturbances (such as emotional or cognitive disorders) in extremely preterm infants and is impossible to detect on ultrasound.

Due to the increased risk of mental disorders, including autism spectrum disorders, it seems important to include these infants in early psychological developmental assessment and psychiatric screening, which will allow early diagnosis and implementation of treatment [18, 19]. There are two types of screening tools for the detection of ASD: level 1 and level 2 screening measures. Level 1 screeners are designed to distinguish children with ASD from typically developing peers. In contrast, level 2 screeners serve to differentiate between the risk of ASD and other developmental disorders. A good screening tool for autism spectrum disorders is still sought for, as the most commonly used the M-CHAT test (a level 1 screener) gives many false-positive results in preterm infants [20, 21]. STAT (Screening Tool for Autism in Toddlers and Young Children, Stone et al., 2000; 2004 ), as a level 2 screening tool, has been recommended by the American Academy of Neurology (Filipek et.al., 2000). STAT is an interactive measure, conducted in a playful and joyful manner, and it only takes 20 to 30 minutes to administer. An important feature of this screener is that it can be used by a wide range of community specialists (psychologists, paediatricians, etc.). Among the few longitudinal, follow-up studies of premature infants’ development that have been carried out in Poland, none, to our knowledge, attempts to investigate the possible interconnections between neuroimaging results and neurodevelopmental outcomes, including the assessment of risk for autism spectrum disorder.

## Aim

The aim of the study was to analyse the relationship between brain imaging results (US and MRI) and neurological, psychological and psychiatric development evaluations of two-year-olds born extremely prematurely.

## Material and methods

The study population were infants born ≤28 GA and hospitalised at NICU between June 1, 2013 and December 31, 2015. We present the results of the first 12 children who were admitted to the study at the age of 2 years (corrected age) and had undergone both neuroimaging, and neurological, psychological and psychiatric assessments.

- Cranial ultrasound was performed early (3-14 day of life, the number of tests varied, depending on the diagnosed pathology) and late (around 38-40 postconceptional weeks). Sonographic examinations were performed using two ultrasound systems: Aloka ProSound Alpha 7 (linear transducer 4-10 MHz, convex transducer 4-11 KHz) and Philips iU-22 (transducers 5-12 MHz and 5-8 MHz, respectively). Anterior and mastoid fontanelles were used.
- Magnetic resonance imaging was performed between 38 and 42 postconceptional weeks with a 1.5T scanner (GE Signa HDxt) using an “adult” sixteen-channel head coil and since the second half of the year 2013 − in the dedicated neonatal eight-channel, phase-array head coil in the MR-compatible incubator (Nomag IC 1.5, Lammers Medical Technology GmbH; INC). MRI protocol included:
  – Coronal T2-weighted images (T2WI),
  – Axial T1-weighted images (T1WI),
  – Axial SWI sequence and GRE/T2*WI,
  – DWI sequence,
  – Sagittal T2WI,
  – Axial T2WI,
  – Axial FLAIR sequence,
  – Coronal FSPGR/3D/T1WI.
- MR venography, arteriography, and other additional sequences, including post-contrast imaging, were performed according to current needs with an individual approach to every patient.
- At 3-6-9-12-18-24 months of corrected age paediatric and neurological evaluation was performed in addition to which psychomotor development assessment was carried out. This was based on the Denver scale and on selected elements of Prechtl’s spontaneous movement assessment at 3 and 6 months. Important medical events that may have influenced CNS development and the time of the introduction and the scope of early stimulation and rehabilitation were recorded. Children were classified into three groups: normal, abnormal, for further observation.
- At 2 years of corrected age, psychological assessment of psychomotor development using *The Children Development Scale (CDS)* [22] was performed, each child’s activity in free-play and semi-structured situations was observed and a clinical interview with the parents was conducted. *The Children Development Scale* is designed to assess psychomotor development of children aged 2 months to 3 years and consists of 10 subtests: *Manipulation*, *Perception*, *Scribble & Drawing*, *Building Blocks*, *Similarities*, *Memory*, *Speech*
- *& language*, *Vocabulary*, *Social behaviour, and Motor skills*. Only the subtests suitable for the child’s age are administered. Composite results for each subtest are computed, and then a global result is calculated with relevant confidence intervals, and interpreted with reference to respective age norms. Thus, the CDS assessment provides information as to both the level and the profile of the child’s psychomotor development.
- At 2 years of corrected age the level 2 screening test STAT (Screening Tool for Autism in Toddlers and Young Children) was administered by a psychiatrist, which was – to our knowledge − the first time that this tool had been used in extremely premature children in Poland. STAT is a direct, play-based assessment of a child’s social-communicative behaviours with a special focus on directing attention, requesting, reciprocal and pretend play, and motor imitation. High specificity, sensitivity, and predictive validity has been reported in 24 to 35-month-old children with developmental delays, later diagnosed with ASD. A score of 2 points and above is considered as a risk for ASD.
- A thorough ophthalmological examination was carried out according to the current scheme during hospitalization; laser photocoagulation treatment and/or intraocular Lucentis has been used in case of advanced forms of retinopathy. An ophthalmological assessment was then performed until the age of two years.
- A screening hearing test was performed during hospitalization and then an ABR hearing test at the age of a few months.

## Preliminary results

Data from the pregnancy period and characteristics of patients are presented in Table I. Gestational age ranged from 23 to 27 weeks of gestation, and birth weight from 580 g to 1100g. The high prevalence of prenatal corticosteroid treatment (83%) and delivery by caesarean section (83%) was noted. Corticosteroid treatment was required in 50% of cases during hospitalization, due to the long duration of mechanical ventilation (mean 22 days). 25% of the children were diagnosed with moderate or severe bronchopulmonary dysplasia (BPD). As many as 75% of the children have suffered from retinopathy of prematurity (ROP), requiring treatment (intraocular Lucentis or laser photocoagulation). Periventricular infarction was diagnosed in 3 children (25%), accompanied by a third degree of bleeding in 2 of them (in 1 case with posthaemorrhagic ventricular dilatation).

**Table I:** Characteristics of the study population. Tabela I. Charakterystyka ogólna badanej populacji.

Population characteristics *Dane ogólne*	Infantsincluded in analysis *Populacja badana* (n=12**)
Gestational age (GA) weeks: median (range) *Wiek* *ciążowy* *(tyg.)*: *średnia* *(zakres)*	26(23+6;27+0)
Birth weight (g): median (range) *Urodzeniowa masa ciała: średnia (zakres)*	795(580-1100)
Singleton n (%) *Ciążą pojedyncza n (%)*	12 (100)
Prenatal steroids n (%) *Kortykosteroidy prenatalnie n (%)*	10 (83)
Caesarean section n (%) *Cięcie cesarskie n (%)*	10 (83)
Boys n (%) *Płeć męska* *n (%)*	8 (67)
Days on mechanical ventilation: median (range) *Długość wentylacji mechanicznej w dniach: średnia(zakres)*	22 (1;60)
Postnatal steroids n (%) *Kortykosteroidy postnatalnie n (%)*	6 (50)
BPD (O_2_ at GA of 36 weeks) n (%) BPD (*O_2_ w 36 tyg WP) n (%) *	3(25)
IVH grade III n (%) *IVH III stopnia n (%)*	0
PHI n (%) *Zawał krwotoczny n (%)*	2 (17)
IVH grade III / PHI n (%) *IVH III stopnia/Zawał krwotoczny n (%)*	2 (17)
NEC n (%) *Martwicze zapalenie jelit n (%)*	1 (8)
ROP requiring treatment n (%) *ROP wymagająca leczenia n (%)*	9 (75)

Definition of abbreviations: *Definicja użytych skrótów*: BPD − bronchopulmonary dysplasia; IVH − intraventricular haemorrhage; PHI − periventricular haemorrhagic infarctions; NEC − necrotising enterocolitis *BPD – dysplazja oskrzelowo-płucna, IVH – krwawienie około-dokomorowe, PHI* − *zawał krwotoczny, ROP – retinopatia wcześniacza*

Table II summarizes brain imaging, neurological evaluation, assessment of psychomotor development, and screening for autism spectrum disorders in all individuals. Normal ultrasound scans and brain MR images, normal neurological examination and psychomotor development were found in 4 children (33.3%).

**Table II:** Neuroimaging and neurologic examination and psychomotor development (n=12). Tabela II. Neuroobrazowanie a badanie neurologiczne i rozwój psychoruchowy populacji badanej (n=12).

Lp	Initials *Inicjały*	Gestational age (GA), weeks *Wiek ciɋżowy (a tyg.)*	US at term-adjusted age *USG p/ciemiɋczkowe w okolicach terminu porodu*	MRI at term-adjusted age *MR mózgu w okolicach terminu porodu*	Neurological exam 2 years corrected age *Badanie neurologiczne w wieku korygowanym 2 lat*	Psychomotor development assessment 2 years corrected age *Rozwój psychoruchowy w wieku korygowanym 2 lat*	STAT assessment 2 years corrected age *Badanie STAT w wieku korygowanym 2 lat*
1	HB	26+0	normal *prawidłowe*	normal *prawidłowe*	normal *prawidłowe*	average ** or above na poziomie *przeciętnym i powyżej*	negative *ujemne*
2	KW	26+0	IVH II, posthemorragic dilatation *IVH II, PPK*	IVH II, posthemorragic dilatation *IVH II, PPK*	normal *prawidłowe*	normal *prawidłowy*	no data *brak danych*
3	MD	26+0	normal *prawidłowe*	IVH II cerebellar haemorrhage *IVH II, krwawienie do móżdżku*	normal *prawidłowe*	Iow *niski*	positive *dodatnie*
4	NL	26+0	IVH II	IVH II, posthaemorrhagic cerebellar injury /destruction *IVH II, pokrwotoczna destrukcja móżdżku*	for further observation *do dalszej obserwacji*	*general delay globalne* *opóźnienie*	*positive dodatnie*
5	00	24+6	normal *prawidłowe*	IVH II, intra- and pericerebellar haemorrhage *IVH II, krwawienie do- i wokół móżdżku*	Cerebral palsy (CP) *Mózgowe porażenie dziecięce*	general delay *globalne opóźnienie*	no data *brak danych*
6	PJ	26+1	normal *prawidłowe*	lll/PHI, cerebellar haemorrhage *IVH lll/zawał krwotoczny, krwawienie do móżdżku*	normal *prawidłowe*	general delay *globalne opóźnienie*	positive *dodatnie*
7	RO	24+0	IVHIII/PHI>5mm *IVH III Zawał krwotoczny >5 mm*	IVH lll/PHI>5mm, parietal lobe *IVH Ill/Zawał krwotoczny >5 mm, płat ciemieniowy*	ataxia, observation for *CP/ataksja, do obserwacji* w *kierunku MPDz*	normal *prawidłowy*	negative *ujemne*
8	SK	26+0	** normal prawidłowe	*normal prawidłowe*	*normal prawidłowe*	*normal prawidłowy*	negative *ujemne*
9	MK	28+0	normal *prawidłowe*	focal haemorrhage in frontal lobe *punktowe krwawienie* w *płacie czołowym*	normal *prawidłowe*	normal *prawidłowy*	positive *dodatnie*
10	DM	26+2	** normal prawidłowe	*normal prawidłowe*	*normal prawidłowe*	*normal prawidłowy*	negative *ujemne*
11	ZW	27+0	*normal prawidłowe*	*normal prawidłowe*	*normal prawidłowe*	*normal prawidłowy*	negative *ujemne*
12	SD	23+6	** normal prawidłowe	IVH 1, cerebellar *haemorrhage IVH* *I, krwawienie do móżdżku*	ataxia, for further *observation ataksja*, *do dalszej obserwacji*	*general delay globalne* *opóźnienie*	negative *ujemne*

Definition of abbreviations: *Definicja użytych skrótów*: IVH - intraventricular haemorrhage; PHI - periventricular haemorrhagic infarctions; CP - Cerebral palsy *IVH - krwawienie około-dokomorowe, PHI - zawał krwotoczny, MPDz - mózgowe porażenie dziecięce, PPK - pokrwotoczne poszerzenie komór*

### 1 Neuroimaging: comparison of cranial ultrasound scans and brain MR images

The ultrasound examination performed between 38 and 42 weeks post-conception showed no abnormalities in 9 children (75%), whereas MRI performed at the same time showed normal results in 4 children (33.3%), only. The brain was normal in both techniques in 4 children (33.3%). Abnormalities in both techniques and similar lesions were found in 2 cases among the remaining eight patients. Additional information found on MRI were cerebellar abnormalities in 5 cases (hemispheric haemorrhage in 4, pericerebellar haemorrhage in 1, including one case with post-haemorrhagic cerebellar hemisphere destruction diagnosed with MRI and not visible on ultrasound), and in one case a small post-haemorrhagic infarct with 3 mm diameter in the frontal lobe.

### 2 Neuroimaging and neurological and ophthalmic examination

Neurological examination was performed around 2 years of corrected age (between 1 and 10/12 and 2 and 1/12).

It was normal in 8 out of 12 patients (66.7%). There were 4 children with normal neuroimaging (US, MRI) in this group. In the remaining 4 cases, MRI showed grade II intraventricular haemorrhage, also seen on ultrasound, and additionally cerebellar haemorrhage.

In 4 (33.3%) children the neurological examination was abnormal, including 1 with cerebral palsy diagnosis and 2 with ataxia. MRI revealed haemorrhagic infarction and post-haemorrhagic ventricular dilatation in one, as on US, and grade II IVH with cerebellar haemorrhage, not visible on ultrasound, in three patients.

Even though 9 children were diagnosed with ROP requiring treatment, one child only had severe visual impairment due to vision loss in one eye as a grade 5 ROP consequence. All the other children showed good binocular vision.

## 3 Neuroimaging and psychomotor development and screening for autism spectrum disorders

Psychomotor development evaluation and autism spectrum disorder screening were performed around 2 years of corrected age (between 1 and 10/12 and 2 and 1/12).

At least average psychomotor development results were found in 7 of 12 children (58%). In four of them, US and MRI showed no abnormalities, while a post-haemorrhagic ventricular dilatation was observed in 3 children (including 1 case in both neuroimaging methods) and haemorrhagic infarct (2 cases, on MRI only). No abnormalities were found in the cerebellum. It should be stressed that in the group of children with at least an average level of psychomotor development, abnormal results of neurological examination (ataxia, observation for CP) were detected in one patient only, while reduced results in at least two of the Children Development Scale sub-tests were present in 3 children (Motor − in all cases, Speech, Vocabulary, Social Behaviour and Scribbling and Drawing − in two, and Comparison – in one case).

Psychomotor development was assessed as lower than average (low or very low / global developmental delay) in 5 children (42%). Cerebellar or pericerebellar bleeding on MRI was reported in all these cases. Neurological examination was normal in 2 children.

Screening for autism spectrum disorder (STAT) was performed in 10 children (83%). It is important to mention that in all the children who presented the risk of autism spectrum disorder (4/10, i.e. 40%) abnormalities on MRI were detected: cerebellar haemorrhage in 3, frontal lobe post-haemorrhagic infarct in 1 child. Positive screening results were associated with abnormal psychomotor development in 3 children, but with abnormal neurological examination in 1 child only.

## Discussion

The study group is small and cannot provide a basis for statistical analysis, but there is a clear tendency to associate abnormal psychomotor development at the age of two years with abnormal brain MRI findings at term. Abnormalities were related to cerebellar pathology in all the cases. The same applies to screening for autism spectrum disorder. The increased risk of ASD has been reported in most cases with cerebellar haemorrhage. However, this result should be treated with great caution and needs to be verified in further diagnostic tests that will show how many of the children in the risk group actually meet the ASD criteria in further development. Such studies are planned as part of the continuation of this project.

US was normal in 4 cases with abnormalities in at least one field: neurological assessment, psychomotor development and STAT, including a case of cerebral palsy, while MRI was not normal in any of them.

In view of the increasing number of papers devoted to the association of cerebellar bleeding with neurodevelopmental problems of prematurely born children, its increased detection rate on MRI is clinically very meaningful. The superiority of MRI over US in this aspect (and not only this one) is not a new finding and has already been described (e.g. 23). In the recent paper by Parodi et al. it has been stated that microhaemorrhages proved to be undetectable by ultrasound even using mastoid fontanelle [24]. MRI is nowadays regarded as the gold-standard technique for the assessment of brain lesions associated with prematurity with emphasis on cerebellar (micro)haemorrhages. There is already quite a large number of studies (e.g. by Limperopoulos et al.) that link cerebellar damage in the premature brain with secondary underdevelopment of cortical projection regions in the contralateral cerebral hemisphere. Decreased prefrontal grey matter volume has been proven to be significantly associated with social and behavioural impairment [25]. In another recent paper Brossard-Racine and colleagues have proved on Diffusion Tensor Imaging (DTI) that preterm infants, even in the absence of overt cerebellar parenchymal injury detected on conventional MRI, have abnormal cerebellar microstructure reflected by fractional anisotropy (FA) and mean diffusivity (MD) alterations as compared with term-born controls [26]. So our focusing on cerebellar damage detected by MRI is clinically and scientifically justified, as the use of MRI increases our ability of prognostication and may be crucial for early therapy.

The preliminary results of our analysis are consistent with other authors suggesting a relatively high prevalence with an increased risk of autism spectrum disorders in children born prematurely [15, 27]. It should be stressed that the risk of ASD is not the same as definitive ASD clinical diagnosis. Furthermore, many authors point to the particularly high prevalence of prematurely-born babies whose positive screening results for ASD are not confirmed in clinical diagnosis [19]. This is especially true for children with various types of health complications, sensory abnormalities and psychomotor development difficulties [28, 29]. Screening tests may produce false-positive results in such circumstances [27]. An in-depth, psychological and psychiatric assessment of a child’s developmental profile, including the specificities of developmental dificulties and competencies, is therefore essential.

On the other hand, children with ASD symptoms may show results below their actual abilities in developmental scales not because of actual cognitive delay, but because of their dificulty in communicating with the diagnostician, refusal to follow instructions, over-absorbance in other activities, etc. These children, due to their adaptive, social and communication dificulties, o&en refuse to engage in a task, and in developmental assessment based on standardized measures they can achieve results that are lower than their actual development potential.

Significantly, early diagnosis of developmental dificulties, especially in extremely premature babies, cannot be regarded as stable in time. Hence, it is important to monitor development and adapt all forms of prevention, early intervention and treatment to the individual developmental needs of the child. This requires cooperation within a team of paediatrician-neurologist-psychologist-psychiatrist. It may cause problems due to significant difierences in the assessment of child development by difierent professionals. One of the reasons is the above-mentioned variable (time-varying) degree of child interaction with a particular examiner. There is no standardized questionnaire / interview tool for parental assessment of child development in Poland. Parents do not always provide the same information on subsequent visits to different professionals. In addition, specialists in different fields use different, not always compatible research tools. For example, a norm for premature infants (especially for extremely preterm ones) in psychomotor development assessment using the Denver scale (which is a screening tool) is the so-called boundary zone, which is very wide. This norm is accepted, because the destructive role of damage is more distinct in disorganizing orderly sequences of physiological processes than in the destruction of individual brain centres. As a consequence, the functional age of children whose brain was prenatally and perinatally significantly exposed to adverse factors remains far behind the chronological age, regardless of possible neurological deficits (30). In the CDS, which is a standardized tool for the general population, the same results will qualify a child below the norm (low or global delay) (Table II).

It should be emphasized that while the results presented here are preliminary, they have already encouraged a deeper reflection on the developmental trajectory complexity of children with extreme prematurity. The observed variety of possible configurations of neurological, psychological and psychiatric findings illustrates the scale of diagnostic and prognostic challenges in this group of children well, and encourages special caution. The results of the analysis indicate the need to take into account the information about development obtained from difierent sources and using difierent methods. They also encourage a prospective, long-term, multidisciplinary and multi-faceted follow-up of the development of children with extreme prematurity, including neurological, psychological and psychiatric assessments. Moreover, it should be stressed that the effectiveness of therapeutic interventions in the group of post-infancy children, related to the improvement of developmental outcomes, especially the level of the development of social and communication competences, has been confirmed by numerous studies [31, 32].

## Conclusions

Without neglecting the primary role of transfontanelle ultrasound, we emphasize that magnetic resonance imaging is much more eficient in the evaluation of central nervous system lesions in preterm neonates and better correlates with their further development. The visualization of lesions e.g. in the posterior cranial fossa, remains the domain of MRI, although ultrasound is also performed through the mastoid fontanel. The potential association of these changes with abnormal psychomotor development at the age of two years and with autism spectrum disorder which is raised in the literature and indicated by the results of this preliminary report requires further studies and implies the use of MRI at a term-equivalent age.

The preliminary results of the present study indicate the complex relationships between neuroimaging results, neurological findings, and trajectories of psychological development in extreme preterms, requiring confirmation in further research. The functioning diversity of the children examined indicates the need to place extremely premature babies under well-planned, multi-specialty and long-term care which includes their mental development and functioning evaluation.
